# Comparing the COVID-19 pandemic in space and over time in Europe, using numbers of deaths, crude rates and adjusted mortality trend ratios

**DOI:** 10.1038/s41598-021-95658-4

**Published:** 2021-08-12

**Authors:** Valentina Gallo, Paolo Chiodini, Dario Bruzzese, Elias Kondilis, Dan Howdon, Jochen Mierau, Raj Bhopal

**Affiliations:** 1 University of Groningen, Campus Fryslân, Wirdumerdijk 34, 8911 CE Leeuwarden, The Netherlands; 2 Queen Mary University of London, London, UK; 3 London School of Hygiene and Tropical Medicine, London, UK; 4 Medical Statistics Unit, University of Campania “L. Vanvitelli”, Naples, Italy; 5 Medical Statistics, University of Naples “Federico II”, Naples, Italy; 6 Aristoteles University, Thessaloniki, Greece; 7 University of Leeds, Leeds, UK; 8 Aletta Jacobs School of Public Health, University of Groningen, Groningen, The Netherlands; 9 Usher Institute, University of Edinburgh, Edinburgh, Scotland, UK

**Keywords:** Diseases, Medical research

## Abstract

Comparison of COVID-19 trends in space and over time is essential to monitor the pandemic and to indirectly evaluate non-pharmacological policies aimed at reducing the burden of disease. Given the specific age- and sex- distribution of COVID-19 mortality, the underlying sex- and age-distribution of populations need to be accounted for. The aim of this paper is to present a method for monitoring trends of COVID-19 using adjusted mortality trend ratios (AMTRs). Age- and sex-mortality distribution of a reference European population (N = 14,086) was used to calculate age- and sex-specific mortality rates. These were applied to each country to calculate the expected deaths. Adjusted Mortality Trend Ratios (AMTRs) with 95% confidence intervals (C.I.) were calculated for selected European countries on a daily basis from 17th March 2020 to 29th April 2021 by dividing observed cumulative mortality, by expected mortality, times the crude mortality of the reference population. These estimated the sex- and age-adjusted mortality for COVID-19 per million population in each country. United Kingdom experienced the highest number of COVID-19 related death in Europe. Crude mortality rates were highest Hungary, Czech Republic, and Luxembourg. Accounting for the age-and sex-distribution of the underlying populations with AMTRs for each European country, four different patterns were identified: countries which experienced a two-wave pandemic, countries with almost undetectable first wave, but with either a fast or a slow increase of mortality during the second wave; countries with consistently low rates throughout the period. AMTRs were highest in Eastern European countries (Hungary, Czech Republic, Slovakia, and Poland). Our methods allow a fair comparison of mortality in space and over time. These might be of use to indirectly estimating the efficacy of non-pharmacological health policies. The authors urge the World Health Organisation, given the absence of age and sex-specific mortality data for direct standardisation, to adopt this method to estimate the comparative mortality from COVID-19 pandemic worldwide.

## Introduction

In December 2019, a cluster of pneumonia cases in Wuhan City (China) was identified as having been caused by the SARS-CoV-2 virus, leading to the disease now termed COVID-19. The subsequent global transmission led to the outbreak being classified as a pandemic by the World Health Organisation (WHO) on 11th March 2020^1^. Some of the clinical characteristics of COVID-19 infection (long incubation period, heterogeneity of symptoms, transmission by asymptomatic carriers)^1–5^ have contributed to make the estimates of its distribution at population level at the beginning of the pandemic somewhat challenging. Nonetheless, monitoring the pandemic at the national level and comparing data at the international level is of paramount importance. Comprehensive surveillance data, and transparency in the communication with the population are necessary prerequisite for controlling the pandemic in a timely and focused manner and for adjusting healthcare services in a constantly changing environment of dynamic healthcare needs^6–8^. The COVID-19 pandemic has already revealed the structural weaknesses of public health surveillance systems in both high and low-income countries and the difficulty in producing complete and comparable data within and among countries^9–11^. It is therefore essential, when comparing data from different countries, to use only appropriate metrics that rely on complete data collected in a similar way, assuring comparability^12^. Testing is not widely available in every country, including many European countries during the first months of the pandemic; moreover, testing policies differ by country. In countries where testing is less readily available, it is likely that this would lead to undercounts of infected people, especially of those showing fewer symptoms. This has the potential to introduce selection bias, underestimating incidence and inflating case fatality ratios. Mortality, conversely, suffers from less variability given that it is independent from testing policy, and that it should be recorded fairly consistently across countries given the uniqueness of the clinical picture of the people severely affected by COVID-19. This includes a progressive respiratory insufficiency leading to an interstitial pneumonia with rrespiratory deterioration concomitant with extension of ground-glass lung opacities on chest CT scans, lymphocytopenia, and high prothrombin time and D-dimer levels^13^. At individual level, mortality has been shown to be associated with age, sex, ethnicity, socio-economic status, level of deprivation, and outdoor environment, in the United States^14–16^; and with a number of co-morbidities, including hypertension, diabetes mellitus, congestive heart failure, dementia, chronic pulmonary disease, liver disease, renal disease, and cancer^17, 18^.

For international comparisons aimed at monitoring changes in trends over time and in space, adjusting for all risk factors associated to mortality might be very demanding. However, given the particular age- and sex- distribution of morbidity and mortality from COVID-19^4, 19^, the underlying sex- and age-distribution of a population is of particular importance in determining the number of expected cases. Not adjusting for age undermines meaningful comparison especially when comparing lower- with higher-income countries. During the first months of the pandemic, due to its sudden onset and speed of spread, it has been difficult to compare data coming from different countries partly because basic epidemiological principles (e.g. adjustment for age and sex) have not been applied consistently, with the main emphasis given to the number of cases per population unit^12, 20^. Only recently, this matter has gained some more attention^21^.

Standardised Mortality Trend Ratios (SMTRs) were used to describe the COVID-19 related mortality trends over time across Italian regions by some of us^22^. The aim of this paper is to expand on that approach and to present a method for monitoring trends of COVID-19 mortality adjusted for the demographic composition of the underlying population, based on the concept of indirect standardisation^23^. The method is illustrated by an application to data coming from European countries.

## Methods

### Definition used

The definition of a “case” affects the metrics used to monitor trends of COVID-19. Cases can be defined as anyone who has been infected by SARS-CoV-2—“confirmed case”^24, 25^; or anyone who is symptomatic. In the first case, a form of biological test would be needed to detect the infected but asymptomatic cases^24, 25^. The choice of the definition of case is likely to make a large difference in any metric used. The initial estimates that up to 90% of infected people were asymptomatic have been recently revised and estimated to be between 17 and 20%^26^. Nonetheless, it still represents a considerable proportion of people going potentially completely undetected by the health system, in absence of active surveillance. In many contexts, detecting all those infected is complicated given that the availability of serological tests with good sensitivity and specificity for wide-scale use is not uniformly distributed^27, 28^. Ascertaining COVID-19 mortality is also no entirely problem-free; the distinction between *mortality with the infection* (COVID-19 positive deaths, regardless of the immediate cause of death) and *mortality from the infection* (deaths occurring in those for whom the immediate or underlying cause of death can be reasonably ascribed to COVID-19) has not always been applied in a clear-cut way. However, this has improved since the World Health Organisation (WHO) published guidance for the use of deaths *from* COVID-19 for surveillance purposes (“(…) death resulting from a clinically compatible illness, in a probable or confirmed COVID-19 case, unless there is a clear alternative cause of death that cannot be related to COVID-19 disease (e.g. trauma)”^29^.

### Sample, data collection and research setting

This paper uses data readily available online, from different sources, downloaded and collated in an Excel spreadsheet for analysis. Daily total number of deaths from COVID-19 from selected European countries (Austria, Belgium, Czech Republic, Denmark, Estonia, Finland, France, Germany, Greece, Hungary, Iceland, Ireland, Italy, Latvia, Lithuania, Luxembourg, the Netherlands, Norway, Poland, Portugal, Slovakia, Slovenia, Spain, Switzerland, and United Kingdom) from 17/03/2020 to 29/04/2021 were extracted from the European Centre for Disease Prevention and Control (ECDC) website^30^, these were summed up to display cumulative number of deaths per day per country. Age-and sex- distribution of death from COVID-19 reported from subset of these countries was collected from varied national-based sources^30–33^. The demographic composition of the underlying population in 2019 of the selected countries was extracted from the Organisation for the Economic co-operation and Development (OECD)^34^. Crude mortality rate per 1,000,000 was calculated daily in each country by dividing the number of cumulative deaths by the total country population in 2019.

### Methods and data analysis procedure

A reference population and a reference period of time was conveniently defined as the population of the European countries for which the age- and sex-distribution of deaths from COVID-19 was available, before the end of March 2020. A total of 14,086 COVID-19 deaths divided into age and sex categories occurred during the reference period (649 from the United Kingdom -UK- up to 27/03/2020^31^, 4993 from Italy released on 23/03/2020^32^, 821 from Belgium^30^, 3459 from France^33^, 581 from Germany^30^, 187 from Portugal^30^, and 3396 from Spain^30^, all up to 31/03/2020) and composed the reference population. These were collated in one composed age- and sex-specific set of COVID-19 related deaths and used to calculate *age- and sex-specific mortality rates* per 1,000,000 population by dividing for each category the total number of COVID-19 related deaths by the source populations (UK, Italy, Belgium, France, Germany, Portugal, and Spain) of the same age- and sex- specific category. These overall age- and sex-specific mortality rates were applied to the age- and sex- specific distribution of the population of each of the included European countries in order to estimate the *number of expected cases* in each country, which can be interpreted as the number of COVID-19 deaths that each country would have had if they experienced the rates of the reference population on 31/03/2020^23^.

For each day, the number of cumulative observed deaths in each country was divided by the number of expected deaths by end of the reference period, and multiplied by 100 to calculate the Standardised Mortality Trend Ratio (SMTR) in the i-th day, with the following formula, as done previously^22^:

$$SMTR_{i} = \frac{{\left( {Cumulative\, observed\, deaths} \right)_{i} }}{Expected\, deaths\,by\,the\,end\,of\, reference\, period }* 100$$

95% confidence intervals of SMTR were calculated assuming a Poisson distribution. Subsequently, each SMTR was multiplied by the crude death rate calculated in the reference period (total number of COVID-19 deaths in the reference period divided by the total reference population) multiplied by 10,000 in order to obtain the Adjusted Mortality Trend Ratio (AMTR) per million inhabitants in the i-th day, applying the following formula, as described previously^23^:

$$AMTR_{i} = SMTR_{i} \times CMR_{reference\, sample} * 10,000$$

The AMTR can be interpreted as the age- and sex-adjusted number of deaths per million inhabitants due to COVID-19 if the population had experienced the same mortality rate as the reference population in the reference period (January–March 2020).

## Results and discussion

### Cumulative mortality

**Figure 1:**
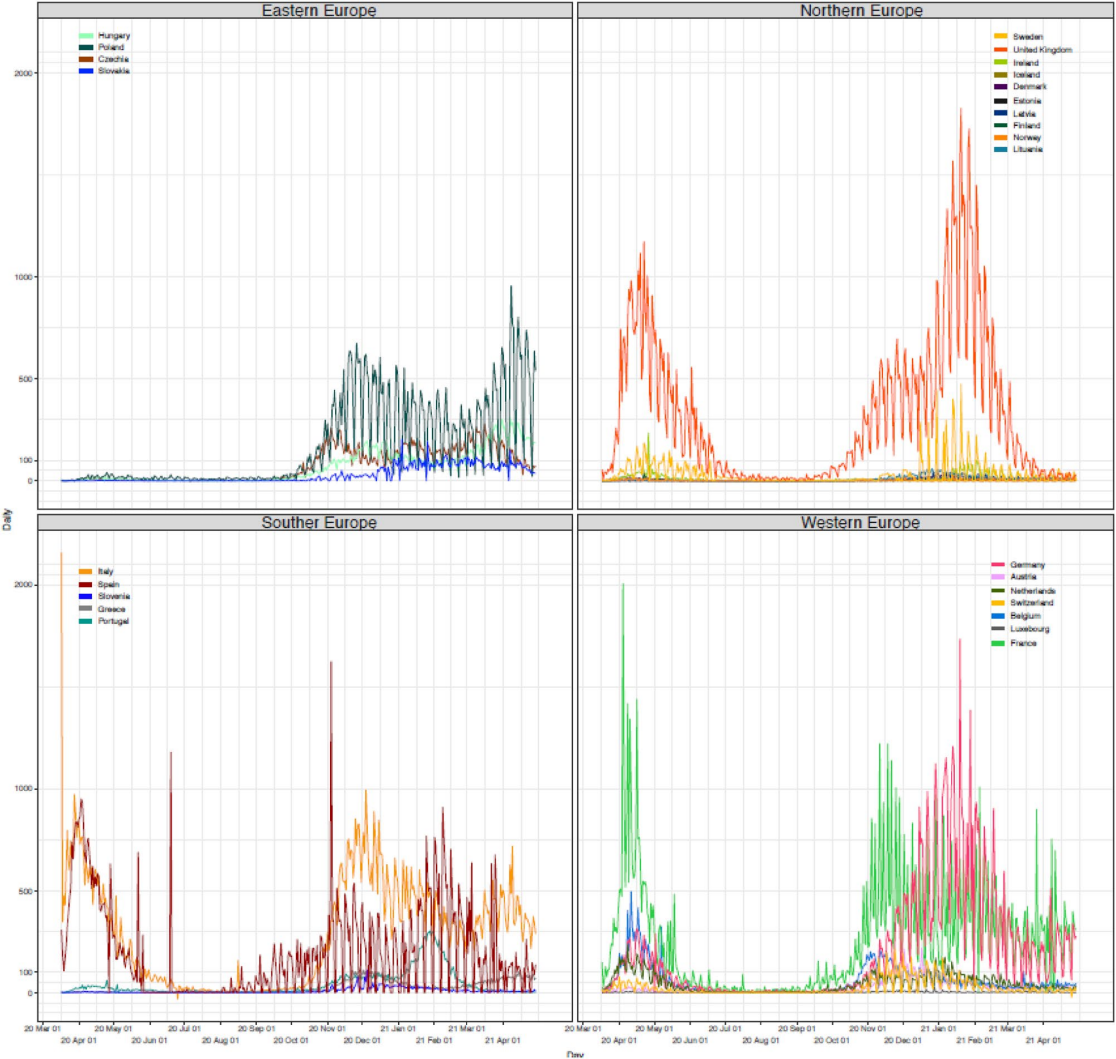
Daily mortality from COVID-19 reported in selected European counties from 17/03/2020 to 29/04/2021.

Daily mortality varied by country and over time, with France reporting the overall highest daily mortality on 04/04/2020, with 2004 deaths from COVID-19 in one day. Daily number of deaths are plotted against time in Fig. 1 by European macro-region, Eastern, Norther, Southern, and Western countries. Daily cumulative numbers of deaths were calculated in all European countries which were then ranked (Table 1).

**Table 1:** Ranking of the included European countries for absolute number of death from COVID-19, crude mortality rates, and Adjusted Mortality Trend Rations (AMTRs) on 20/06/2020.

	Absolute number of deaths from COVID-19 on 29/04/2021	Crude mortality rate from COVID-19 on 29/04/2021	AMTRs from COVID-19 on 29/04/2021
1st	United Kingdom	Hungary	Hungary
2nd	Italy	Czech	Czech
3rd	France	Luxembourg	Slovakia
4th	Germany	Slovakia	Poland
5th	Spain	Belgium	Belgium
6th	Poland	Italy	United Kingdom
7th	Czech	United Kingdom	Slovenia
8th	Hungary	Poland	Luxembourg
9th	Belgium	Slovenia	Spain
10th	Netherlands	Spain	Italy
11th	Portugal	Portugal	France
12th	Sweden	France	Ireland
13th	Slovakia	Lithuania	Portugal
14th	Greece	Sweden	Lithuania
15th	Austria	Switzerland	Sweden
16th	Switzerland	Austria	Latvia
17th	Ireland	Latvia	Switzerland
18th	Lithuania	Ireland	Austria
19th	Slovenia	Netherlands	Netherlands
20th	Denmark	Germany	Estonia
21st	Latvia	Greece	Germany
22nd	Estonia	Estonia	Greece
23rd	Finland	Denmark	Denmark
24th	Luxembourg	Finland	Norway
25th	Norway	Norway	Finland
26th	Iceland	Iceland	Iceland

Overall, a two-wave pattern is immediately evident for most of the early affected countries (UK, Italy, France, Spain, Portugal, Ireland, Luxembourg, Switzerland, Belgium, Sweden, and the Netherlands) with steep increase in mortality starting March/April 2020, a levelling off by the summer, and a new increase by October/November 2020. Hungary, Germany, Poland, Czech Republic, Slovakia, Greece, Lithuania, Latvia, Estonia, Denmark, and Austria, on the other hand, witnessed an increase with varying steepness during the second wave, despite having had very low mortality beforehand. Italy was the first country registering more than 20,000 deaths on 14/04/2020, and totalled the second highest death tool of 120,053 deaths about a year later by the end of the observation period (29/04/2021). A similar pattern was observed in France and Spain, with only about a week’s delay, which however levelled off reaching a total of 104,302 and 79,684 deaths by 29/04/2021, respectively. In Spain the increase mortality during the second wave starting from November 2020 was less pronounced compared to Italy and France. Conversely, in the UK, despite the 20,000 deaths threshold being reached about 10 days after Italy, the death toll increase was steady exceeding other countries by 14/05/2020 and the steep increase in mortality during the second wave was abruptly levelled off starting in February/March 2021 most probably as result of the early implementation of the vaccination campaign^35^. Nonetheless, UK registered the highest toll in Europe with 128,136 deaths by the end of the observation period. Germany and Poland, despite having avoided high mortality during the first waves, witnessed a steep increase of the number of daily deaths starting from November 2020 reaching a total of 82,206 and 66,977 deaths, respectively, by 29/04/2020.

### Crude mortality rates

**Figure 2:**
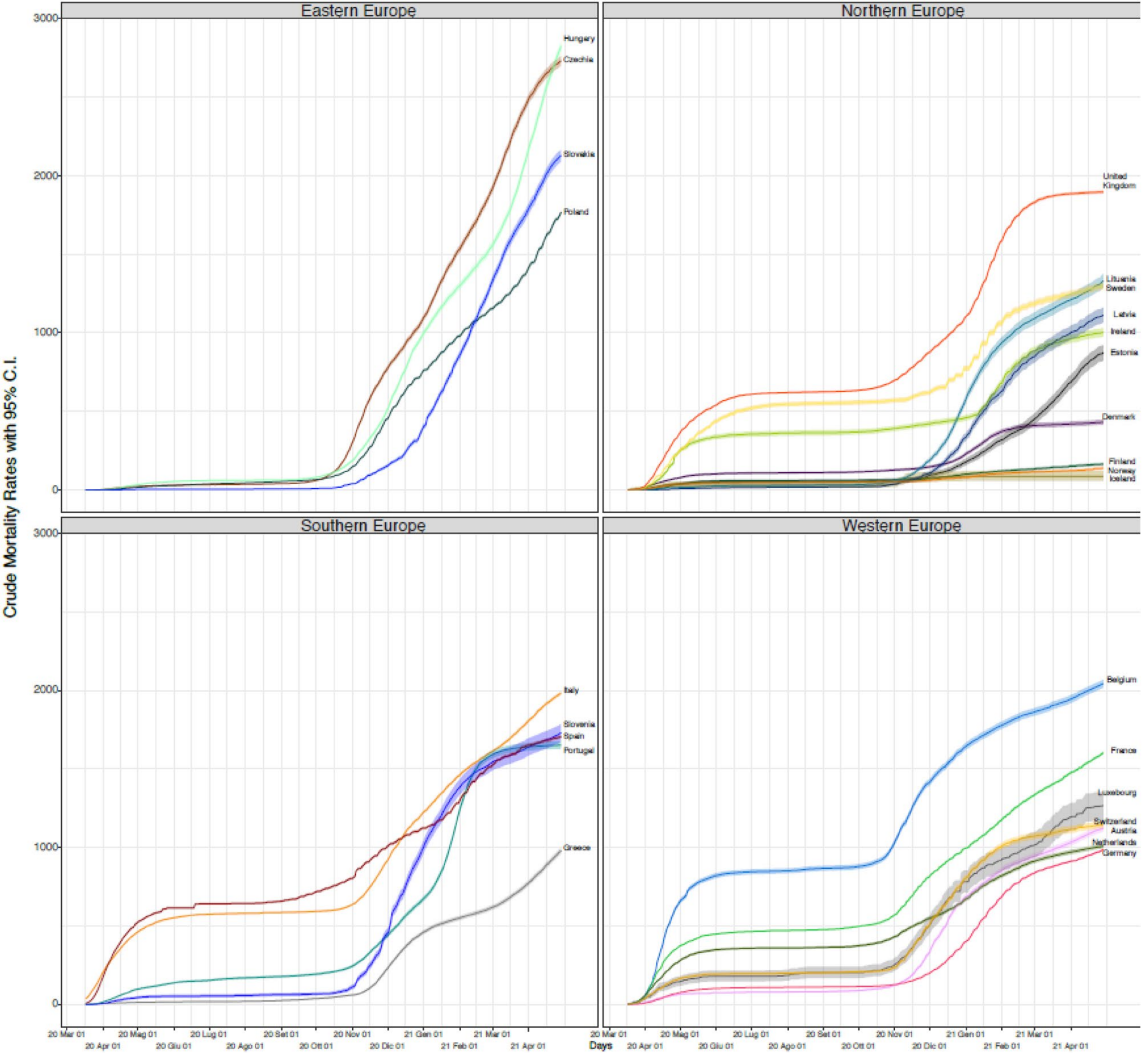
Crude Mortality Rates (CMRs) from COVID-19 per 1,000,000 inhabitants in European countries from 17/03/2020 to 29/04/2021.

Crude mortality rates were plotted as shown in Fig. 2 maintaining the division by macro-region for facilitating interpretation; the resulting ranking of countries by the end of the study period is reported in Table 1. On 29/04/2021, in Europe, the total mortality rate from COVID-19 was 1558 per million inhabitants; this was highest in Hungary (2801 per million inhabitants on the same date) and lowest in Iceland (82 deaths per million inhabitants). Once accounting for the total country population, Hungary, Czech Republic, and to a lesser extent Slovakia, reached the highest crude mortality rates, cumulated only during the second wave, starting around October 2010. Belgium experienced the worst crude mortality rate among the countries going through two waves, followed by Italy, UK, and to a lesser extent France, Spain and Luxembourg. Poland, Slovenia, and Portugal, and to a lesser extent Lithuania reached high crude mortality rates during the second wave only, but kept those below 1800 deaths per million.

### Adjusted mortality trend ratios (AMTRs)

**Figure 3:**
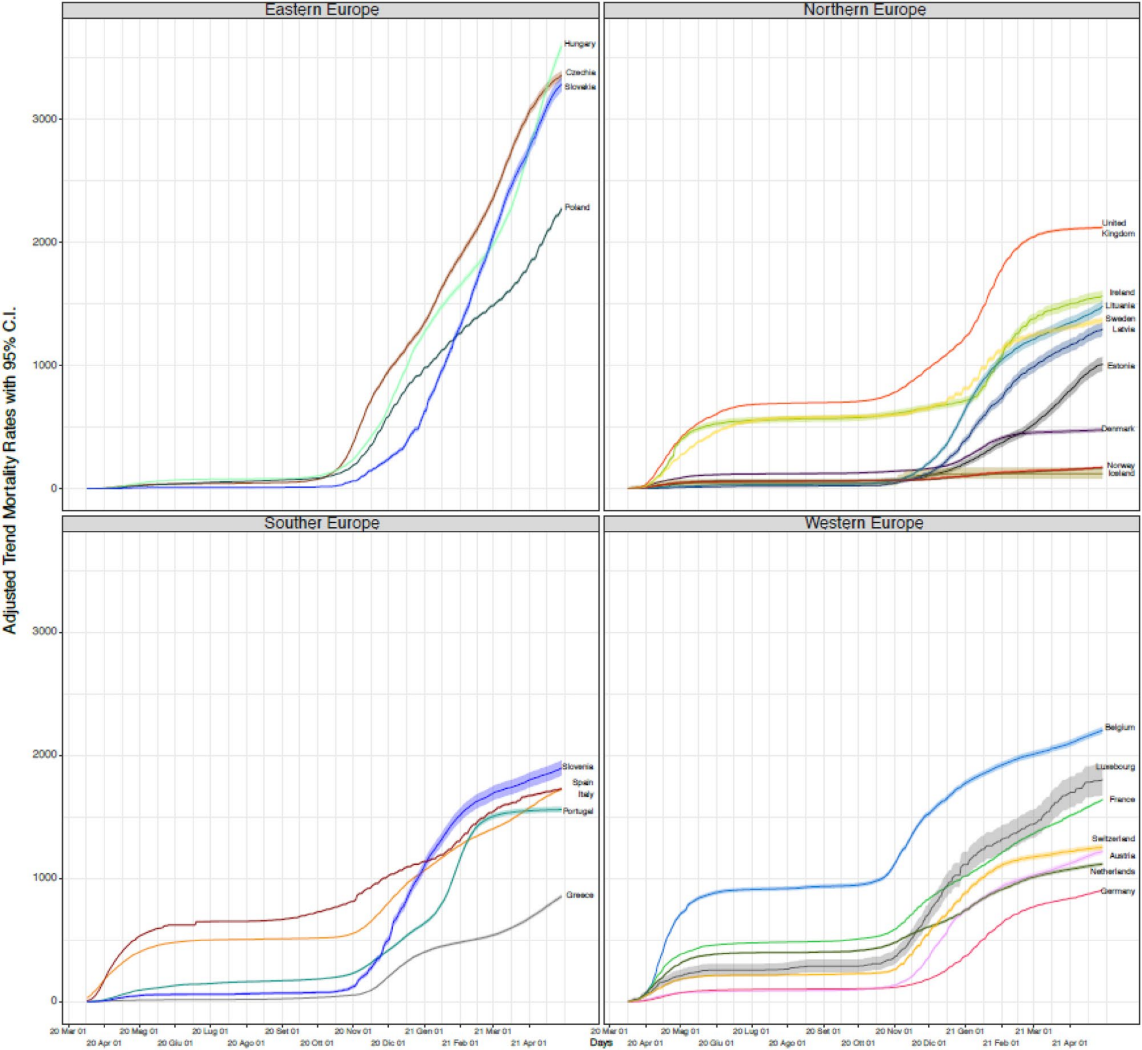
Adjusted Mortality Trend Rates (AMTRs) per 1,000,000 inhabitants due to mortality from COVID-19 in European countries from 17/03/2020 to 29/04/2021.

AMTRs of each country are plotted in Fig. 3, and countries were ranked accordingly in Table 1. When accounting for the underlying age and sex structure of each country, the picture of COVID-19-related mortality distribution in Europe changed further, and four different patterns could be identified: countries which experienced a two-wave pandemic, countries which did not experience the first wave, but saw either a fast or slow increase in mortality during the second wave; and countries which maintained low mortality rates throughout the pandemic. These will be analysed and discussed in details in the following sections.

### Countries experiencing two-wave pandemic

Eleven European countries experienced a clear-cut two-wave pattern, albeit with some differences in intensity: Belgium, UK, Luxembourg, Spain, Italy, France, Sweden, Portugal, Ireland, Switzerland and the Netherlands. Among these, Belgium remained the single country experiencing the highest adjusted mortality rate (AMTR on 29/04/2021: 2204/million), followed by the United Kingdom with a AMTR of 2119/million on the same date. Interestingly, AMTRs in UK have flattened considerably starting from March 2021, probably as result of the early implementation of the vaccination campaign^36^. Once accounting for the age- and sex-distribution of their underlying ageing populations, France, Italy and Spain were comparatively less impacted compared to UK and Belgium, in particular during the second wave, reaching similar AMTRs (1640; 1726; and 1727/million, respectively).

Notably, Luxembourg and Ireland, which had relatively low CMRs, ranked higher, with AMTRs higher than 1500/million (1802 and 1562/million, respectively) due to the young age distribution of their underlying populations. Sweden witnessed a steep increase in mortality during the first wave, comparable to the increase in the UK, probably as result of not implementing and social distancing measures^37^. In this category, the Netherlands succeeded best in keeping the second wave under control, despite e relatively high first wave. By the end of the observation period their AMTRs was 1117.

All these countries belonging to the Western, Southern and Northern European groups played an important role in international trade and were the ones with the highest COVID-19 importation risk during the early days of the panemic due to their high travel flows directly from China^38^. Several factors might explain why these relatively richer countries with mature healthcare systems were so heavily affected by the pandemic, in addition to geography and positioning in the global economy^39^. Fiscal decentralisation and fragmented public health policy responses between regions might also have contributed explaining low pandemic preparedness and performance in countries like Belgium, Spain and Italy^39, 40^. Early evidence from Italy, for example, showed how lack of efficient data and information exchange between regional public health authorities undermined the country’s ability to respond to the pandemic in a timely manner^41^. Privatisation and fragmentation of epidemiological surveillance systems offers and additional explanation of the inability of this group of countries to effectively control the pandemic^39^. Evidence suggested for example that the outsourcing of UK’s test and tracing system led to significant data gaps and undermined local authorities ability to control the pandemic in an effective and focused manner^42^.

### Countries with fast rising mortality during the second wave only

Hungary, Czech Republic, Slovakia, and Poland, and to a lesser extent Slovenia, reported much higher age- and sex-adjusted mortality rates than the other European countries, with the highest AMTRs, despite having experienced very low rates during the first wave. Hungary remained the country experiencing the highest mortality, with AMTR of 3593/million by 29/04/2021, with this steep mortality rise manifested only during the second wave. COVID-19-related mortality in Slovakia was very similar to that experienced in the Czech Republic, once accounting for the age- and sex-distribution of the underlying population. Both countries reached an ATMR of 3245 and 3357/million respectively by the end of the observation period. In line with these countries, Poland and Slovenia, which succeeded in shielding the population from the first wave, experienced similar rapid increase of mortality during the second wave, albeit less steep compared to Hungary, Czech Republic and Slovakia. AMTRs for Poland by the end of the observation period was 2274/million, and for Slovenia 1897/million.

Although there is no single and uniform explanation for this group of countries, their early success in containing the first wave of the COVID-19 pandemic is most probably related to the timely introduction of social isolation measures and their relatively limited exposure to imported cases due to their peripheral role in global economy and international trade^39^. It is also worth noticing that the Eastern European countries have weak public health and healthcare systems, as reflected to their significantly lower share of public revenues to total health expenditure compared to EU average^43^, a fact that probably explains their poor performance during the second wave of the pandemic when SARS-CoV2 had already significantly spread in the community. Authoritarian political leadership, and controversial-populistic policy responses combined with an ill resourced health care system is the most probable explanation of Hungary’s low pandemic preparedness performance during the second wave of the pandemic^44^.

### Countries with slow rising mortality during the second wave only

Latvia, Lithuania, Austria, Estonia, Germany, Denmark and Greece are countries which experienced a relatively low mortality during the first wave (with AMTRs not exceeding 250/million), and a relatively slow increase in mortality during the second wave.

This group of countries is quite heterogenous consisting of high-income central European countries, peripheral European economies (such as Greece) and Baltic countries. With the exception of Germany all of these countries faced low COVID-19 importation risk^38^, which could explain their good performance during the first wave of the pandemic. Regarding their pandemic preparedness and their performance during the second pandemic wave, country-specific analysis would be needed in order to understand national trends and draw country-specific conclusions. For example Greece, heavily affected by the 2008–18 recession and austerity was ill prepared for a public health threat of such magnitude; the vulnerabilities of its public healthcare services and surveillance system were fully revealed when cases and deaths steeply increased during the second and third wave of the pandemic in the country despite an almost six months lasting second national lockdown^45^.

### Countries with consistently low-COVID-19-related mortality

Finland, Iceland, and Norway are the countries, among the included, which experienced a consistently low COVID-19 related mortality reaching an a AMTR of 166, 119, and 174/million, respectively. In these Nordic countries high social cohesion, relatively lower social inequalities, higher average public expenditure on health and social protection compared to other European countries are the most probable explanations of their relatively better performance during the COVID-19 pandemic^43, 46^. Although Iceland, contrary to other islandic countries like New Zealand, kept its borders open to tourism, it seems to have counterbalanced the risk of imported cases by extensive testing of its population and double-testing of tourists during both waves of the pandmic^47^.

### Overall strengths and limitations of this methodological approach

This paper provides a simple method to compare COVID-19 mortality trends over time and across countries, accounting for the underlying age structure of populations. By only using the total number of COVID-19 related deaths, routinely collected in many countries, this method allows the calculation of AMTRs which are useful to comparatively monitor pandemic trends, not only when comparing areas with profoundly different age structure, such as the global North and the global South^48^, but even comparing different European countries. Most reports on COVID-19 epidemiological data to date have failed to take the underlying population structures into account. Some of the previous attempts performed a standardisation on case fatality rates^49–51^ therefore not overcoming the problems of possible bias due to local policies, despite the standardisation. Only Heuveline and Tzen used an approach comparable to the present one, through an indirect standardisation^21^; however they provided only a method for cross-sectional comparisons, and derived their sex- and age- specific ratios from the US population. This might represent a problem as age- and sex- specific mortality might vary across countries, for example according to the different prevalence of co-morbidities^52^ which increase the risk of mortality in COVID-19 positive individuals^53^. Conversely, using AMTRs, standardised against the age- and sex-distribution of mortality in a reference population coming from the same countries under study, during a reference period, allowed a comparison of trends both in space and over time uncovering features of the pandemic otherwise not easily detectable. In countries where the social distancing measures have not been taken (i.e. Sweden) or have been substantially delayed (i.e. Hungary) the curve of AMTRs shows a much steeper shape compared to other European countries which have enforced stricter rules.

Deriving the age- and sex-specific rates from the sample population which is part of the population to which data is standardised, guarantees consistency of the estimate allowing for differences in the distribution of underlying risk factors, and in the overall performance of the national health systems to be accounted for and not to artificially bias the estimates, as can happen when rate are derived from external standard populations. Caution needs to exerted in interpreting the AMTRs: given that COVID-19 crude fatality rate changes over time^50, 54^, AMTRs cannot be easily extrapolated to estimate infection prevalence over time.

The main potential limitations of this approach refers to the definition of mortality for COVID-19. Discrepancies in defining deaths from COVID-19 at country/region level would affect the number of deaths reported and therefore the estimated AMTRs. If all authorities would follow the strict guidance provided by the WHO^29^, discrepancies will be minimised reinforcing the reliability of the present method. For example, some scientists have shared concerns on how mortality from COVID-19 is being ascertained in Belgium where people dying in care homes have been classified as dying from COVID-19 based on indirect evidence (the presence of infection in the care home and the reporting of compatible symptoms)^55^. In addition, by calculating the cumulative mortality, any error in death reporting would be carried on in the analysis. Moreover this method assumes that mortality remains constant over time in the given populations. However, it is likely that the age-specific mortality rates calculated during the first phases of the infection reflect an increased mortality of a more vulnerable population exposed to comorbidities. Finally, it is important to bear in mind that mortality does not solely reflect the spread of disease: mortality from COVID-19 is a function of disease incidence, severity, and quality of healthcare systems to cope with infected and diseased people. Some have argued that patters of cohabitations, which are strongly associated with the age structure of population in some cases, can influence discrepancies between infection and mortality. A modelling study predicted in Italy a higher mortality with less infections compared to Mozambique which would experience more infections but lower mortality, once adjusted for age^56^. If confirmed, this would hamper even more the monitoring of infection through mortality.

### Practical implications and future directions

Reliable comparisons of impact of COVID-19 at regional or national level are essential for the monitoring of the spread of diseases in space and over time, and are instrumental for the retrospective analysis of different non-pharmacological policies currently enforced in many affected countries^57, 58^. Identifying four different patterns of COVID-19-related mortality distribution prompted the analysis of similarities and differences in national policies which can contribute identifying the main drivers of the pandemic mortality. In a context of rapid production of scientific evidence aimed at contributing to the understanding and management of a pandemic of devastating proportion such as the COVID-19 one, the release of solid, comparable data should be a high priority. The method proposed in this paper allows comparisons of mortality in space and over time. By calculating age- and sex-distribution of mortality during a reference period in wide populations (i.e. per continent), it would be possible to immediately and reliably compare the burden of COVID-19 mortality across countries only plugging in the cumulative number of deaths (as reported by Our World in Data, for example^59^). The authors urge the WHO to adopt this method to estimate the burden of COVID-19 pandemic worldwide given the lack of international data by age and sex that can permit direct standardised rates. This would enrich the currently available data visualisation tools^30, 59, 60^ with values better suited for international comparison across demographically diverse regions of the world in order to indirectly evaluate the effect of pharmacological and non-pharmacological interventions, and the environmental risk of exposure^61^. Notably, these comparison are now a days more and more important for both predicting the insurgence of new variants via epidemiological surveillance, and the efficacy of the vaccine campaigns, at population level.

## Data Availability

All data used for this manuscript is publicly available on the referenced websites. Dr Gallo had full access to all of the data in the study and takes responsibility for the integrity of the data and the accuracy of the data analysis. She declares that this manuscript is honest, accurate, and transparent account of the study being reported; that no important aspects of the study have been omitted. All co-authors had full access to the data, and can take responsibility for the integrity of the data and the accuracy of the data analysis.
